# Efficacy of 4% 5-Fluorouracil Cream in the Treatment of Actinic Keratoses: A Single-Center Experience

**DOI:** 10.3390/jcm15020612

**Published:** 2026-01-12

**Authors:** Carmen Cantisani, Antonio Di Guardo, Giovanni Paolino, Natasa Balázs, Mehdi Boostani, Norbert Kiss, Claudio Conforti, Francesca Feresin, Andrea Carugno, Luca Gargano, Luigi Losco, Steven Paul Nisticò, Giovanni Pellacani

**Affiliations:** 1 UOC of Dermatology, Department of Clinical and Cardiovascular Sciences, “Sapienza” University of Rome, 00185 Rome, Italy; antonio.diguardo@uniroma1.it (A.D.G.); feresinfrancesca@gmail.com (F.F.); lucagargano1995@gmail.com (L.G.); steven.nistico@gmail.com (S.P.N.); pellacani.giovanni@gmail.com (G.P.); 2 IDI-IRCCS, Dermatological Research Hospital, 00167 Rome, Italy; c.conforti@unilink.it; 3 Unit of Dermatology, IRCCS San Raffaele Hospital, 20132 Milan, Italy; gio8519@gmail.com; 4 Department of Dermatology, Venereology and Dermatooncology, Semmelweis University, 1085 Budapest, Hungary; natasabalaz75@gmail.com (N.B.); mehdi_parsii@yahoo.com (M.B.); norbert.f.kiss@gmail.com (N.K.); 5 Department of Dermatology, Roswell Park Comprehensive Cancer Center, Buffalo, NY 14203, USA; 6 Department of Medicine and Surgery, University of Insubria, 21100 Varese, Italy; andrea.carugno@uninsubria.it; 7 Department of Medicine, Surgery and Dentistry, University of Salerno, 84081 Baronissi, Italy; luigi.losco@gmail.com

**Keywords:** actinic keratosis, 5 fluorouracil, local skin reactions, non-melanoma skin cancer

## Abstract

**Background/Objectives:** Actinic keratoses (AKs), also known as solar keratoses, are considered premalignant skin lesions that can evolve into squamous cell carcinoma (SCC). Among the available options, 5-fluorouracil (5-FU) remains a cornerstone. **Methods:** This study is a retrospective analysis of our database of the non-melanoma skin cancer outpatient clinic. The main objective was to evaluate patients treated with 4% 5-FU cream for AK lesions. The efficacy of 4% 5-FU was evaluated retrospectively by measuring the percentage of patients who achieved complete clearance. A secondary efficacy measure was the percentage of partial clearance, defined as at least a 75% reduction in lesion count. Additionally, the study aimed to assess the safety of 4% 5-FU cream. **Results:** We included 150 patients clinically diagnosed with AK, treated with 4% 5-FU cream and evaluated 432 lesions. Complete clearance of lesions was observed in 138 patients (92%) with partial clearance in 12 patients (8%). At 12 months, the recurrence rate was 11%. **Conclusions:** Based on our analysis, 4% 5-FU cream is an effective and well-tolerated treatment for AKs, particularly in patients with extensive field cancerization. While local skin reactions are a natural part of its mechanism, they are manageable and do not outweigh clinical benefits.

## 1. Introduction

Actinic keratoses (AKs), also known as solar keratoses, are among the most common premalignant skin lesions worldwide [1,2]. Their incidence continues to rise, making them a significant public health concern due to their potential to progress into invasive squamous cell carcinoma (iSCC). The prevalence of AKs increases with age and cumulative ultraviolet (UV) exposure, particularly in fair-skinned individuals, affecting up to 60% of older adults in high-UV regions and approximately 20–30% in Europe [3]. AKs develop from chronic and cumulative UV exposure, particularly in fair-skinned individuals (Fitzpatrick I–III), who are more vulnerable to photodamage [4,5,6,7,8,9]. UV radiation induces direct DNA damage, oxidative stress, and immunosuppression, leading to clonal expansion of dysplastic keratinocytes. Mutations in tumor suppressor genes, particularly TP53, play a central role in this process, driving the progression from subclinical alterations to clinically visible AKs and, in some cases iSCC [10].

As a pyrimidine analog antimetabolite, 5-FU inhibits thymidylate synthase, disrupting DNA synthesis and inducing apoptosis in dysplastic keratinocytes [11,12]. 5-FU has a dual mechanism of action, it does not just inhibit thymidylate synthase and block DNA synthesis, but its incorporation into RNA disrupts ribosomal processing, mRNA maturation, and tRNA function. In keratinocytes, this RNA toxicity is a major driver of cell death, possibly more important than DNA effects [13]. Rapidly dividing atypical keratinocytes in AKs are more vulnerable to 5-FU than normal epidermal cells, explaining its efficacy and field-directed utility [14].

Daylight photodynamic therapy with 5-ALA or MAL offers high efficacy and excellent cosmetic results, particularly for large or cosmetically sensitive areas [15]. Diclofenac has a favorable tolerability profile but relatively modest clearance rates, while imiquimod stimulates local immune responses and is particularly useful on the face and scalp. Tirbanibulin has gained attention as a novel, short-course regimen with good efficacy and tolerability, showing favorable outcomes in network meta-analysis (OR ~11.1 vs. placebo) and in phase III trials in comparison to vehicle.

Randomized controlled trials, including head-to-head studies such as Krawtchenko et al., demonstrated clinical clearance rates of 96% for 5-FU, 85% for imiquimod, and 68% for cryotherapy; one-year sustained clearance was 54% for 5-FU, 73% for imiquimod, and 28% for cryotherapy [16]. Notably, in network meta-analysis, topical 5-FU formulations (both 4% and 5%) demonstrated the strongest odds of complete clearance compared with placebo (OR ~30.3 and ~35.0, respectively), supporting its continued role as the most effective and cost-efficient field-directed option. This combination of efficacy, long-term protective effect, and availability in various formulations underpins its continued role as the cornerstone of AK management. First approved in 1962, 5-FU remains widely used in various topical formulations (0.5–5%), with evidence from numerous randomized trials supporting its superiority over placebo and many comparators.

Its efficacy is independent of the inflammatory reaction [17].

By sharing our clinical experience with 5-FU and reviewing the literature, this study aims to provide insights into lesion selection, treatment response, and the management of adverse effects in patients with AKs.

## 2. Materials and Methods

This was a retrospective observational study conducted at the non-melanoma skin cancer outpatient clinic of Policlinico Umberto I Hospital, Rome, Italy. All consecutive eligible patients fulfilling the inclusion criteria were included; no formal sample size calculation was performed. Clinical images stored in the patient database were assessed to evaluate lesion progression, treatment response, and overall disease management. Data were collected from adult patients with AKs who had been treated with commercially available 4% 5-FU cream once-daily for four weeks between January 2022 and January 2023, and who had at least one follow-up visit documented. Follow-up visits were routinely performed at approximately 3, 6, and 12 months. AK lesion clearance was assessed clinically, by experienced dermatologists based on characteristic clinical features, and dermoscopically by comparing the findings at each follow-up with baseline photographs. No high-definition imaging device was used. The clinical and dermoscopic differentiation between actinic keratosis (AK) and squamous cell carcinoma in situ (SCC in situ) followed the European consensus-based interdisciplinary guideline for diagnosis and treatment of actinic keratoses [18]. According to this guideline, AK was identified by superficial scaling, background erythema, and characteristic dermoscopic patterns such as the strawberry pattern, white scales, and visible follicular openings. SCC in situ was diagnosed based on the presence of glomerular vessels, more confluent erythema, and diffuse scaling. No subtyping of AK lesions (e.g., Bowenoid, hypertrophic, pigmented variants) was performed. Biopsy was not routinely performed and was reserved only for lesions with dermoscopic features suspicious for early invasion. Treatment response was categorized as complete clearance (complete disappearance of the lesion), partial clearance (visible reduction in lesion size or thickness), or no response (no clinically relevant improvement). Biopsy was not routinely performed and was reserved only for lesions with clinical or dermoscopic features suspicious for SCC. All patients had provided written informed consent as part of routine clinical care.

Patients were eligible if they had grade I or II AKs according to the three-point Olsen scale, with lesions located on the face, ears, and/or scalp. A smaller subset of patients with SCC in situ or AKs on the dorsal hands were also included when treated with the same regimen. Only visible and palpable lesions were included, while hypertrophic or hyperkeratotic lesions were excluded. Hyperkeratotic lesions were defined as Olsen grade III. These lesions were excluded because hyperkeratotic actinic keratoses are generally not suitable for treatment with topical 5-fluorouracil due to limited drug penetration and poor therapeutic response. Therefore, no hyperkeratotic AKs were treated with 4% 5-FU in our cohort.

The primary efficacy outcome was the proportion of patients achieving complete (100%) clearance of all lesions within the application area at four months. The secondary efficacy outcome was partial clearance, defined as a ≥75% reduction in lesion count at four months. In addition, the secondary safety outcomes included the evaluation of local skin reactions (LSR, local skin reaction) and treatment tolerability. LSR scores ranged from 0 to 3 and comprised six variables: erythema, flaking/scaling, crusting, swelling, vesiculation/pustulation, and erosion/ulceration (0 = absent, 1 = mild, 2 = moderate, 3 = severe). Safety assessments also included documentation of pigmentation or scarring and recording of adverse events. Treatment response was retrospectively evaluated using follow-up macroscopic images, allowing objective assessment of lesion progression and therapeutic outcomes over time.

Furthermore, recurrence at 12 months was included as an additional secondary outcome to provide long-term clinical information.

The study was conducted in accordance with the principles of the Declaration of Helsinki. The study protocol was approved by the Ethics Committee of Sapienza University of Rome, approval number: n.24/2025.

## 3. Results

**Figure 1:**
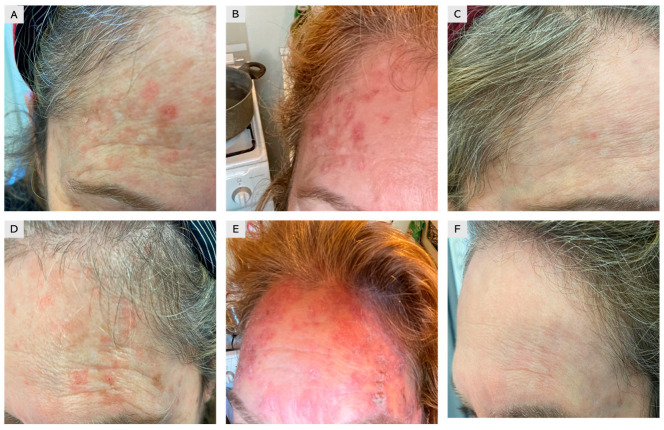
A 52-year-old female patient with multiple actinic keratoses (Olsen I–II) affecting the forehead. (**A**–**C**) Right side: (**A**) baseline image showing multiple lesions; (**B**) severe local skin reaction with erythema and scaling after two weeks of treatment with 4% 5-fluorouracil cream; (**C**) complete healing with favorable cosmetic outcome. (**D**–**F**) Corresponding images of the left side of the forehead showing a similar clinical course.

**Figure 2:**
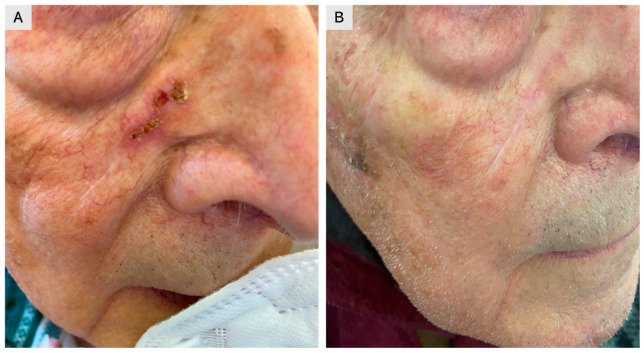
An 86-year-old male patient with squamous cell carcinoma in situ arising in the right nasolabial fold over a surgical scar. (**A**) Baseline image before treatment. (**B**) Post-treatment image at four months, showing complete clearance after application of 4% 5-fluorouracil cream.

**Figure 3:**
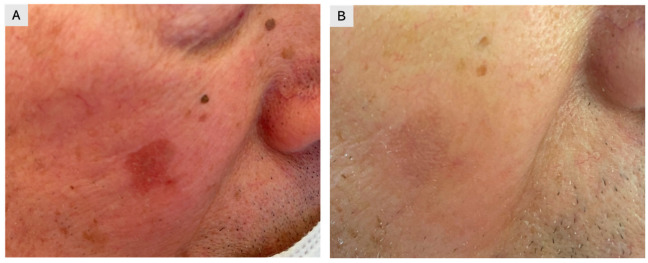
A 61-year-old male patient with actinic keratosis (Olsen I) of the right cheek. (**A**) Baseline image. (**B**) Post-treatment image showing complete resolution of the lesion after application of 4% 5-fluorouracil cream.

**Figure 4:**
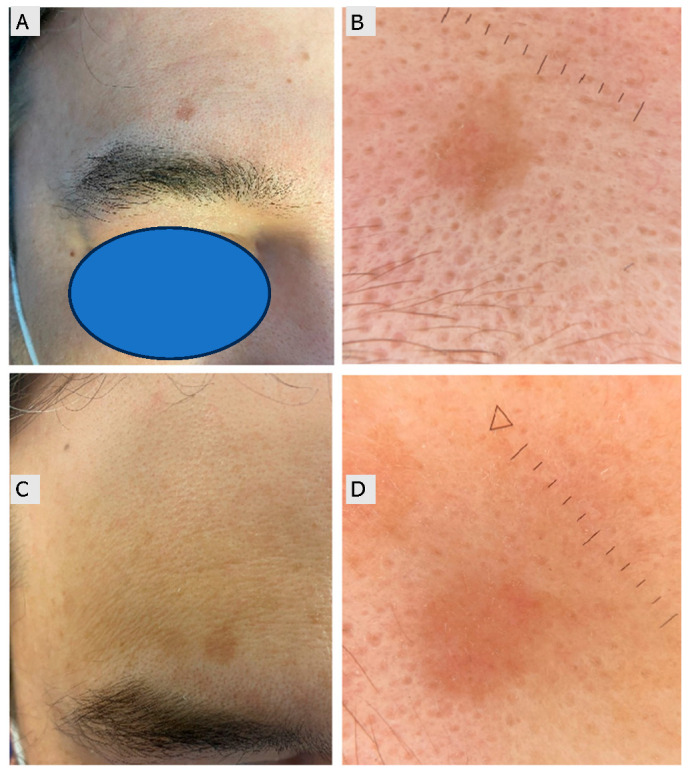
A 45-year-old male patient with a persistent actinic keratosis (Olsen grade I–II) on the forehead. (**A**,**B**) Baseline macroscopic and dermoscopic images. (**C**,**D**) Follow-up images four months after treatment with 4% 5-fluorouracil (5-FU) cream demonstrating minimal clinical and dermoscopic improvement, with persistence of erythema and scaling indicating incomplete response (The triangle and dotted line indicate the measurement scale).

A total of 150 patients clinically diagnosed with single or multiple AKs and treated with 4% 5-FU cream once daily were retrospectively evaluated. Treatment was self-administered for four weeks, and clinical outcomes were assessed using follow-up images stored in our database (Figure 1,Figure 2,Figure 3). The cohort included 94 males (63%) and 56 females (37%), with a mean age of 67 years (range: 45–84) (Table 1). A total of 432 lesions were treated, including 356 AKs (82%) and 76 SCC in situ lesions (18%). Most lesions were located on sun-exposed areas, including the face (53%), scalp (38%), and dorsal hands (19%). At four months, 138 patients (92% of the cohort) achieved complete clearance of all lesions, while 12 patients (8%) achieved partial clearance, defined as a reduction of at least 75% in lesion count Complete clearance was achieved in 92.0% (138/150; 95% CI 86.5–95.4), while partial clearance was observed in 8.0% (12/150; 95% CI 4.6–13.5). At 12 months, recurrence was observed in 11% of patients, predominantly in areas with extensive field cancerization. The 12-month recurrence rate was 10.7% (16/150; 95% CI 6.7–16.6). Local skin reactions were common and expected, most frequently erythema (89%), scaling (78%), and mild-to-moderate discomfort (56%). Severe adverse reactions were rare (2%), limited to localized ulceration. Erythema occurred in 89.3% (134/150; 95% CI 83.4–93.3), scaling in 78.0% (117/150; 95% CI 70.7–83.9), and mild-to-moderate discomfort in 56.0% (84/150; 95% CI 48.0–63.7). Severe adverse reactions were rare, occurring in 2.0% (3/150; 95% CI 0.7–5.7), and were limited to localized ulceration. As complete numeric local skin reaction (LSR) scores were not consistently recorded for all patients, reactions were categorized into mild, moderate, or severe based on available documentation. Most reactions were mild to moderate, typically characterized by erythema and scaling, whereas severe LSRs were rare and transient (Table 2). Patients were advised on supportive care measures, such as the use of zinc oxide and boric acid for approximately 10 days, to improve tolerability. To provide a more comprehensive clinical overview, representative cases of treatment failure and recurrence were also included (Figure 4).

**Table 1:** Baseline characteristics of the study population.

Characteristic	Value
Patients (n)	150
Age (years)	Mean 67 (range 45–84)
Sex	Male: 94 (63%) Female: 56 (37%)
Total lesions	432

**Table 2:** Clinical outcomes and adverse events in patients treated with 4% 5-fluorouracil.

Clinical Outcome	n/N	%
Complete clearance	138/150	92.0%
Partial clearance	12/150	8.0%
12-month recurrence	16/150	10.7%
**Adverse Events** **(Local Skin Reactions)**	**n/N**	**%**
Erythema	134/150	89.3%
Scaling/flaking	117/150	78.0%
Mild–moderate discomfort	84/150	56.0%
Severe reactions (ulceration)	3/150	2.0%

## 4. Discussion

Actinic keratosis (AK) represents a common manifestation of chronic ultraviolet-induced damage and is considered an early stage of the keratinocyte carcinoma spectrum. Understanding this continuum is essential when interpreting treatment outcomes and recurrence dynamics. Clinically, AKs appear as rough, scaly, erythematous, or sometimes pigmented patches or plaques on sun-exposed areas such as the face, scalp, forearms, and hands [19]. They are often more easily felt than seen, with patients describing a sandpaper-like texture. The Olsen classification categorizes AKs into three grades based on lesion thickness [20]. However, some lesions show direct dermal invasion by atypical basal clones (Olsen I), making morphology alone insufficient to predict risk of iSCC [21,22]. Transformation rates vary widely, from 0.025% to 20% per lesion per year, depending on lesion number, patient age, immune status, and prior nonmelanoma skin cancer (NMSC) [23,24]. The concept of field cancerization highlights the importance of treating not only clinically evident AKs but also photodamaged surrounding skin, which may harbor subclinical lesions [25,26]. Non-invasive diagnostic tools such as dermoscopy, HFUS, non-linear microscopy [27,28], reflectance confocal microscopy (RCM), dynamic optical coherence tomography (D-OCT), and line-field OCT (LC-OCT) can support lesion assessment and treatment planning [29,30,31,32]. Therapeutic strategies for AK are generally divided into lesion-directed and field-directed approaches, with the choice depending on lesion morphology, anatomical site, patient preference, and tolerability. Lesion-directed therapies include cryotherapy, curettage with or without electrosurgery, and laser ablation, all of which provide immediate clearance and are most effective for isolated or hyperkeratotic lesions [33,34,35,36]. Cryotherapy with liquid nitrogen remains one of the most widely used procedures, inducing rapid freezing, necrosis, and subsequent immune activation, with reported clearance rates varying widely from approximately 39% to over 83%, depending on freezing time, technique, and lesion characteristics [37,38]. For patients with multiple lesions and widespread photodamage, field-directed treatments are preferable as they address both clinically visible and subclinical lesions. Among topical agents, 5-FU, imiquimod, diclofenac gel, and the recently introduced agent tirbanibulin represent the main options, while procedural approaches such as photodynamic therapy (PDT), chemical peels, and dermabrasion provide additional alternatives [39,40,41,42]. This retrospective study demonstrated the high efficacy and manageable safety profile of 4% 5-FU cream in patients with multiple AKs and field cancerization, with high complete clearance rates at four months and low recurrence at twelve months. These results reinforce the role of 5-FU as a field-directed therapy, particularly in high-risk patients presenting with widespread or aggressive lesions [43]. AKs pose a significant clinical challenge due to their potential progression into iSCC if left untreated [21,44]. As summarized in the Introduction, their pathogenesis relates to chronic ultraviolet-induced DNA damage and immune dysregulation, which supports early evaluation and treatment of both clinical and subclinical lesions. Preventive measures, including regular sunscreen use and photoprotection, remain crucial [45,46,47,48]. Among available therapies, 5-FU remains a cornerstone of field-directed management, providing effective lesion clearance while addressing subclinical field cancerization [49]. Although local skin reactions are common, they are usually manageable and may correlate with response [50]. Our findings are consistent with prior clinical observations (Figure 4). Combination regimens, such as 5-FU with photodynamic therapy, may further improve outcomes in selected patients [51,52]. Artificial intelligence-based image recognition [53,54] and non-invasive imaging techniques, such as D-OCT and LC-OCT, may help personalize treatment decisions and monitor responses [55,56]. Prognosis is influenced by the baseline lesion count, which predicts both treatment outcomes and the risk of recurrence [57].

It is important to note that, beyond the difference in concentration, the 4% 5-fluorouracil formulation used in our study differs from the older 5% product in its vehicle composition. The previously marketed 5% formulation was a gel, whereas the 4% preparation is a cream, resulting in distinct physicochemical properties that may influence percutaneous absorption, local tolerability, and patient adherence. Notably, the 5% gel is no longer commercially available in our country, further underscoring the clinical relevance of evaluating the 4% cream in contemporary practice.

Topical 5-FU has been extensively evaluated in the management of AKs, and multiple randomized controlled trials and meta-analyses consistently confirm its superior efficacy compared to most other field therapies. In a Bayesian network meta-analysis by Wu et al. (2019) [11], which synthesized data from 11 randomized controlled trials including 2256 patients, 5% 5-FU cream was shown to achieve a complete patient clearance rate of 56.8%, substantially outperforming other modalities such as 0.5% 5-FU with salicylic acid (35.7%), 3% diclofenac (6.6%), and cryosurgery (0.9%). The same study also demonstrated that in terms of lesion count reduction, 5% 5-FU cream had a 98.6% probability of being the most effective intervention.

In a comprehensive network meta-analysis by Ezzedine et al. (2021) [58], which evaluated 75 randomized controlled trials reported across 151 publications in immunocompetent adults with head-region AKs, various field-directed therapies, including 4% and 5% 5-FU formulations, were quantitatively compared using outcomes such as complete and partial lesion clearance, as well as withdrawals related to adverse events as a proxy for tolerability. This analysis confirmed that 5-FU formulations were the most efficacious interventions overall; notably, the recently approved 4% 5-FU demonstrated efficacy comparable to the 5% formulation while achieving a more favorable acceptability profile [58].

The more recently introduced 4% 5-FU formulation has also been directly compared to the traditional 5% cream. A pooled analysis conducted by Stockfleth et al. (2022) [24] demonstrated that 4% 5-FU ranked highest in partial clearance and second only to 5% 5-FU in complete clearance, while showing a lower risk of withdrawal due to adverse events. Their findings suggest that the 4% concentration may offer a better balance between efficacy and tolerability, supporting its growing role in routine clinical use.

Askew et al. (2009) [59], in a comprehensive meta-analysis, had earlier reported very high lesion clearance rates with 5% 5-FU, reaching 93.8% at 24 weeks and up to 98.0% at 4 weeks, which were clearly superior to outcomes observed with imiquimod or diclofenac. In the same study, patient-level complete clearance rates averaged 49% with 5% 5-FU, compared with 34.8% for 0.5% 5-FU and 54.5% for imiquimod, again underlining the superior efficacy of 5-FU. Importantly, they also showed significantly higher odds of complete clearance with 5-FU versus cryotherapy, with an odds ratio of 10.8 [59].

The Cochrane review and network meta-analysis by Gupta et al. (2013) further consolidated these findings, reporting that 5-FU, both at 0.5% and 5% concentrations, was among the most efficacious therapies for achieving complete clearance, outperforming cryosurgery, diclofenac, and placebo [60].

A health economic evaluation by Jansen et al. (2020) also highlighted the cost-effectiveness of 5-FU compared with other interventions, suggesting that its high efficacy and durability contribute to reduced recurrence and fewer repeated interventions, which is particularly relevant in healthcare systems with constrained resources [61].

In a comprehensive literature review, Shoimer et al. (2019) [62] evaluated the safety and tolerability profiles of various topical agents used for AKs, with a focus on treatment discontinuation due to severe local site reactions (LSRs). The analysis revealed that while 5% 5-FU was generally well tolerated, lower-dose formulations, particularly 0.5% 5-FU with salicylic acid, were associated with higher rates of discontinuation, reaching up to 9.1% in some studies. The authors emphasized that although these formulations maintain high efficacy, the severity of inflammatory responses remains a limiting factor in real-world adherence. The study also compared LSR rates across alternative field therapies, including imiquimod, ingenol mebutate, and diclofenac, further illustrating that tolerability is a critical factor influencing patient compliance and long-term treatment success [62].

Tolerability continues to be a key determinant of the clinical success of topical field-directed therapies for AK. In their systematic review, Balcere et al. (2019) [63] investigated the prevalence of severe local site reactions (LSRs), associated treatment discontinuation rates, and risk factors for LSRs across various topical field therapies used on the face and scalp. The authors found that severe LSRs manifesting as marked erythema, scaling, crusting, or erosion, are common and can significantly impair patients’ social functioning, thereby reducing adherence to therapy. Across multiple agents, notable rates of discontinuation were reported, driven primarily by the intensity of inflammatory reactions. Furthermore, Balcere et al. identified specific patient-centered and treatment-related risk factors that predispose individuals to more severe reactions, underscoring the importance of tailored therapeutic decisions to optimize tolerability and adherence in clinical practice [63].

This complements the qualitative findings of Singh et al. (2023) [64], whose patient-reported outcomes revealed that treatment-related discomfort such as pain, erythema, and visible skin damage frequently led to premature cessation of therapy or reluctance toward retreatment. Together, these studies highlight the necessity of balancing therapeutic efficacy with tolerability, as well as the importance of individualized care strategies and patient education to ensure sustained adherence [64].

A recent systematic review by Patel et al. (2024) [65] evaluated the comparative efficacy of topical field-directed therapies for AK, analyzing 20 randomized controlled trials. The authors assessed the strength of clinical recommendations for each agent based on available evidence. Topical 0.5% 5-FU and 0.5% 5-FU combined with 10% salicylic acid received the strongest recommendation (grade A), indicating consistent high-quality evidence supporting their use. Diclofenac sodium received a grade B recommendation, while other topical agents, including calcipotriol/5-FU combinations, imiquimod, tirbanibulin, and photoprotection-based regimens, were assigned a grade C, reflecting less consistent evidence.

The review emphasizes the clinical value of 5-FU–based therapies as first-line treatments for field cancerization, reinforcing their role in effectively targeting both visible and subclinical lesions in photodamaged skin [65].

This observation is supported by Sinclair et al. (2021) [66], who reviewed long-term outcomes and noted that while 5-FU and photodynamic therapy provided the highest short-term clearance rates on face and scalp lesions, recurrence remained substantial, with lesion counts at one year returning to approximately half of baseline values. This underscores the chronic and recurrent nature of AK field cancerization and the need for ongoing surveillance and preventive strategies, including photoprotection and early retreatment [66].

Collectively, the available evidence consistently supports topical 5-FU as the most effective field-directed treatment for AK. Multiple systematic reviews and meta-analyses, including those by Wu et al. (2019), Jansen et al. (2020), Askew et al. (2009), and Gupta et al. (2013) [11,59,60,61], rank 5-FU above other commonly used agents such as diclofenac, imiquimod, and ingenol mebutate, as well as cryotherapy. The introduction of the 4% formulation provides an additional tool that appears to retain the efficacy of the 5% cream while improving tolerability. At the same time, variability in patient outcomes due to baseline lesion burden, adherence, and tolerability issues highlights the importance of individualized therapy and close follow-up. Importantly, these findings reinforce the role of 5-FU not only as a first-line treatment but also as a cornerstone of long-term AK management strategies aimed at reducing recurrence and preventing progression to iSCC.

The European Association of Dermato-Oncology (EADO), in collaboration with the European Dermatology Forum (EDF), the European Academy of Dermatology and Venereology (EADV), and the Union of Medical Specialists, issued an interdisciplinary S3 guideline emphasizing that AKs should be regarded as keratinocyte intraepidermal neoplasia warranting active intervention rather than observation, given the unpredictability of progression to invasive disease [18]. This consensus guideline highlights the need to assess not only isolated lesions but also surrounding photodamaged skin for features such as erythema, telangiectasia, pigmentary alterations, atrophy, and the characteristic rough texture, which collectively define field cancerization. Advanced diagnostic techniques, including dermoscopy, reflectance confocal microscopy, and line-field confocal optical coherence tomography, are endorsed as valuable adjuncts to distinguish AKs from invasive tumors and to guide treatment planning. Therapeutic recommendations prioritize field-directed interventions, with topical 5-FU, imiquimod, and photodynamic therapy as cornerstone modalities, acknowledging the role of patient immune status, lesion severity, and treatment tolerability in clinical decision-making. Complementing this European framework, the National Comprehensive Cancer Network (NCCN) in the United States addresses AK management within its guidelines for SCC, identifying AKs and in situ carcinoma as precursor lesions that necessitate treatment to mitigate malignant progression [67]. The NCCN similarly endorses cryotherapy for isolated lesions alongside field-directed treatments such as 5-FU and imiquimod, as well as daylight photodynamic therapy, while explicitly noting that these modalities are not appropriate once invasion has occurred.

Both EADO and NCCN stress the importance of photoprotection and patient education to reduce recurrence and promote long-term disease control. Together, these guidelines converge on the principle that AKs should be managed proactively and comprehensively, supporting the rationale for the present investigation into the efficacy and safety of field-directed treatment strategies such as 4% 5-FU cream [68].

In our cohort, 4% 5-FU cream achieved high clearance with good tolerability, supporting its role as a first-line treatment for field cancerization.

### 4.1. Limitations

This study has several limitations. The retrospective design and single-center setting may limit generalizability, while the absence of a control arm precludes direct comparison with other therapies. Another limitation is the lack of subgroup analysis by anatomical site or Fitzpatrick phototype. Larger, prospective studies are needed to confirm these findings and to further refine patient selection for 5-FU–based treatment strategies.

### 4.2. Future Directions

More recently, a 4% once-daily formulation received approval from the European Medicines Agency in 2020, offering a simplified regimen of up to four weeks [69,70,71,72,73]. Lower-concentration creams (0.5% and 1%) have been developed to reduce local irritation while preserving therapeutic efficacy. Early clinical and preclinical evidence suggests a synergistic effect of 5-FU when combined with calcipotriol, enhancing lesion clearance via activation of CD4^+^ T cell–mediated antitumor immunity (e.g., thymic stromal lymphopoietin induction) and achieving dramatic reduction in AK counts compared to 5-FU alone [74]. Additionally, preliminary case reports and reviews imply that combining 5-FU with imiquimod may be more effective than either agent alone, hinting at complementary cytotoxic and immunomodulatory mechanisms [75].

In recent years, several novel topical 5-fluorouracil (5-FU) delivery systems have been developed to improve percutaneous absorption and tolerability. Nanoparticle-based vehicles and lipid-derived carriers such as liposomes, niosomes, ethosomes, transferosomes, and phytosomes can facilitate deeper drug penetration into actinic keratosis lesions while minimizing irritation of surrounding healthy skin. Early in vitro and pilot clinical studies have shown encouraging results regarding enhanced bioavailability and improved cosmetic outcomes [76]. However, further research is warranted to validate their long-term efficacy, safety, and cost-effectiveness before integration into routine dermatologic practice.

## 5. Conclusions

In conclusion, based on our study, topical 4% 5-FU cream is an effective and generally well tolerated treatment for AKs and SCC in situ. Its once-daily regimen and manageable safety profile support its use as a practical first-line field therapy.

## Data Availability

Data available on request from the authors.

## References

[1] Naldi L., Cassalia F. (2024). Actinic keratosis epidemiology: The good, the bad and the ugly. Br. J. Dermatol..

[2] Eder J., Prillinger K., Korn A., Geroldinger A., Trautinger F. (2014). Prevalence of actinic keratosis among dermatology outpatients in Austria. Br. J. Dermatol..

[3] Fargnoli M.C., Altomare G., Benati E., Borgia F., Broganelli P., Carbone A., Chimenti S., Donato S., Girolomoni G., Micali G. (2017). Prevalence and risk factors of actinic keratosis in patients attending Italian dermatology clinics. Eur. J. Dermatol..

[4] Cantisani C., Paolino G., Melis M., Faina V., Romaniello F., Didona D., Cardone M., Calvieri S. (2016). Actinic Keratosis Pathogenesis Update and New Patents. Recent Pat. Inflamm. Allergy Drug Discov..

[5] Berman B., Cockerell C.J. (2013). Pathobiology of actinic keratosis: Ultraviolet-dependent keratinocyte proliferation. J. Am. Acad. Dermatol..

[6] Werner R.N., Sammain A., Erdmann R., Hartmann V., Stockfleth E., Nast A. (2013). The natural history of actinic keratosis: A systematic review. Br. J. Dermatol..

[7] Lee J.H., Kim Y.H., Han K.D., Park Y.M., Lee J.Y., Park Y.G., Lee Y.B. (2018). Incidence of Actinic Keratosis and Risk of Skin Cancer in Subjects with Actinic Keratosis: A Population-based Cohort Study. Acta Derm Venereol..

[8] Yaldiz M. (2019). Prevalence of actinic keratosis in patients attending the dermatology outpatient clinic. Medicine.

[9] George C.D., Lee T., Hollestein L.M., Asgari M.M., Nijsten T. (2024). Global epidemiology of actinic keratosis in the general population: A systematic review and meta-analysis. Br. J. Dermatol..

[10] Thamm J.R., Schuh S., Welzel J. (2024). Epidemiology and Risk Factors of Actinic Keratosis. What is New for The Management for Sun-Damaged Skin. Dermatol. Pract. Concept..

[11] Wu Y., Tang N., Cai L., Li Q. (2019). Relative efficacy of 5-fluorouracil compared with other treatments among patients with actinic keratosis: A network meta-analysis. Dermatol. Ther..

[12] Calzavara-Pinton P., Calzavara-Pinton I., Rovati C., Rossi M. (2022). Topical Pharmacotherapy for Actinic Keratoses in Older Adults. Drugs Aging.

[13] Kanamaru R., Wakui A. (1988). Mechanism of action of anti-cancer drugs from the viewpoint of RNA metabolism. Gan To Kagaku Ryoho.

[14] Hartinger J., Veselý P., Šíma M., Netíková I., Matoušková E., Petruželka L. (2017). 5-fluorouracil Toxicity Mechanism Determination in Human Keratinocytes: In vitro Study on HaCaT Cell Line. Prague Med. Rep..

[15] Kurwa H.A., Yong-Gee S.A., Seed P.T., Markey A.C., Barlow R.J. (1999). A randomized paired comparison of photodynamic therapy and topical 5-fluorouracil in the treatment of actinic keratoses. J. Am. Acad. Dermatol..

[16] Krawtchenko N., Roewert-Huber J., Ulrich M., Mann I., Sterry W., Stockfleth E. (2007). A randomised study of topical 5% imiquimod vs. topical 5-fluorouracil vs. cryosurgery in immunocompetent patients with actinic keratoses: A comparison of clinical and histological outcomes including 1-year follow-up. Br. J. Dermatol..

[17] Breza T., Taylor J.R., Eaglstein W.H. (1976). Noninflammatory destruction of actinic keratoses by fluorouracil. Arch. Dermatol..

[18] Kandolf L., Peris K., Malvehy J., Mosterd K., Heppt M.V., Fargnoli M.C., Berking C., Arenberger P., Bylaite-Bučinskiene M., Del Marmol V. (2024). European consensus-based interdisciplinary guideline for diagnosis, treatment and prevention of actinic keratoses, epithelial UV-induced dysplasia and field cancerization on behalf of European Association of Dermato-Oncology, European Dermatology Forum, European Academy of Dermatology and Venereology and Union of Medical Specialists (Union Européenne des Médecins Spécialistes). J. Eur. Acad. Dermatol. Venereol..

[19] Reinehr C.P.H., Bakos R.M. (2019). Actinic keratoses: Review of clinical, dermoscopic, and therapeutic aspects. An. Bras. Dermatol..

[20] Olsen E.A., Abernethy M.L., Kulp-Shorten C., Callen J.P., Glazer S.D., Huntley A., McCray M., Monroe A.B., Tschen E., Wolf J.E. (1991). A double-blind, vehicle-controlled study evaluating masoprocol cream in the treatment of actinic keratoses on the head and neck. J. Am. Acad. Dermatol..

[21] Fernández-Figueras M.T., Carrato C., Sáenz X., Puig L., Musulen E., Ferrándiz C., Ariza A. (2015). Actinic keratosis with atypical basal cells (AK I) is the most common lesion associated with invasive squamous cell carcinoma of the skin. J. Eur. Acad. Dermatol. Venereol..

[22] Fernandez Figueras M.T. (2017). From actinic keratosis to squamous cell carcinoma: Pathophysiology revisited. J. Eur. Acad. Dermatol. Venereol..

[23] Dianzani C., Conforti C., Giuffrida R., Corneli P., Di Meo N., Farinazzo E., Moret A., Rizzi G.M., Zalaudek I. (2020). Current therapies for actinic keratosis. Int. J. Dermatol..

[24] Stockfleth E., Bégeault N., Delarue A. (2022). The Overall Number of Actinic Keratosis Lesions Is Not Predictable by the Number of Visible Lesions: Consequences for Field-Directed Therapies. Curr. Ther. Res. Clin. Exp..

[25] Willenbrink T.J., Ruiz E.S., Cornejo C.M., Schmults C.D., Arron S.T., Jambusaria-Pahlajani A. (2020). Field cancerization: Definition, epidemiology, risk factors, and outcomes. J. Am. Acad. Dermatol..

[26] Nart I.F., Cerio R., Dirschka T., Dréno B., Lear J., Pellacani G., Peris K., de Casas A.R., Progressing Evidence in AK (PEAK) Working Group (2018). Defining the actinic keratosis field: A literature review and discussion. J. Eur. Acad. Dermatol. Venereol..

[27] Kiss N., Haluszka D., Lőrincz K., Gyöngyösi N., Bozsányi S., Bánvölgyi A., Szipőcs R., Wikonkál N. (2019). Quantitative analysis on ex vivo nonlinear microscopy images of basal cell carcinoma samples in comparison to healthy skin. Pathol. Oncol. Res..

[28] Boostani M., Wortsman X., Pellacani G., Cantisani C., Suppa M., Mohos A., Goldust M., Pietkiewicz P., Bánvölgyi A., Holló P. (2025). Dermoscopy-guided high-frequency ultrasound for preoperative assessment of basal cell carcinoma lateral margins: A pilot study. Br. J. Dermatol..

[29] Boostani M., Pellacani G., Wortsman X., Suppa M., Goldust M., Cantisani C., Pietkiewicz P., Lőrincz K., Bánvölgyi A., Wikonkál N.M. (2025). FDA and EMA-approved noninvasive imaging techniques for basal cell carcinoma subtyping: A systematic review. JAAD Int..

[30] Boostani M., Bozsányi S., Suppa M., Cantisani C., Lőrincz K., Bánvölgyi A., Holló P., Wikonkál N.M., Huss W.J., Brady K.L. (2025). Novel imaging techniques for tumor margin detection in basal cell carcinoma: A systematic scoping review of FDA and EMA-approved imaging modalities. Int. J. Dermatol..

[31] Varga N.N., Boostani M., Farkas K., Bánvölgyi A., Lőrincz K., Posta M., Lihacova I., Lihachev A., Medvecz M., Holló P. (2024). Optically Guided High-Frequency Ultrasound Shows Superior Efficacy for Preoperative Estimation of Breslow Thickness in Comparison with Multispectral Imaging: A Single-Center Prospective Validation Study. Cancers.

[32] Bozsányi S., Boostani M., Farkas K., Hamilton-Meikle P., Varga N.N., Szabó B., Vasanits F., Kuroli E., Meznerics F.A., Lőrincz K. (2023). Optically Guided High-Frequency Ultrasound to Differentiate High-Risk Basal Cell Carcinoma Subtypes: A Single-Centre Prospective Study. J. Clin. Med..

[33] Vicente A.M., Rius I.O., Gil L.A. (2024). Actinic Keratosis in Solid Organ Transplant Recipients: A Medical Literature Review. Actas Dermo-Sifiliogr..

[34] Cantisani C., Paolino G., Pellacani G., Didona D., Scarno M., Faina V., Gobello T., Calvieri S. (2016). MAL Daylight Photodynamic Therapy for Actinic Keratosis: Clinical and Imaging Evaluation by 3D Camera. Int. J. Mol. Sci..

[35] Del Regno L., Catapano S., Di Stefani A., Cappilli S., Peris K. (2022). A Review of Existing Therapies for Actinic Keratosis: Current Status and Future Directions. Am. J. Clin. Dermatol..

[36] Kiss N., Farkas K., Tosti G., De Gado F., Bergler-Czop B., Fazia G., Tammaro A., Cantisani C. (2022). Photodynamic Therapy with 5-Aminolevulinic Acid Patch for the Treatment of Actinic Keratosis. J. Clin. Med..

[37] Clebak K.T., Mendez-Miller M., Croad J. (2020). Cutaneous Cryosurgery for Common Skin Conditions. Am. Fam. Physician.

[38] Arisi M., Pisani E.G., Calzavara-Pinton P., Zane C. (2022). Cryotherapy for Actinic Keratosis: Basic Principles and Literature Review. Clin. Cosmet. Investig. Dermatol..

[39] Ibrahim S.F., Brown M.D. (2009). Actinic keratoses: A comprehensive update. J. Clin. Aesthet. Dermatol..

[40] Heppt M.V., Dykukha I., Graziadio S., Salido-Vallejo R., Chapman-Rounds M., Edwards M. (2022). Comparative Efficacy and Safety of Tirbanibulin for Actinic Keratosis of the Face and Scalp in Europe: A Systematic Review and Network Meta-Analysis of Randomized Controlled Trials. J. Clin. Med..

[41] Paradisi A., Bocchino E., Mannino M., Gualdi G., D’amore A., Traini D.O., Peris K. (2025). The State of the Art in the Treatment of Actinic Keratosis and Field Cancerization: A Narrative Review. J. Pers. Med..

[42] Blauvelt A., Kempers S., Lain E., Schlesinger T., Tyring S., Forman S., Ablon G., Martin G., Wang H., Cutler D.L. (2021). Phase 3 Trials of Tirbanibulin Ointment for Actinic Keratosis. N. Engl. J. Med..

[43] Pariser D.M. (2022). Approaches to Field Therapy for Actinic Keratoses: Relating Clinical Trial Results to Real-world Practice-A Commentary. J. Clin. Aesthet. Dermatol..

[44] Marks R., Rennie G., Selwood T. (1988). Malignant transformation of solar keratoses to squamous cell carcinoma. Lancet.

[45] Christensen S.R. (2018). Recent advances in field cancerization and management of multiple cutaneous squamous cell carcinomas. F1000Research.

[46] Joura M.I., Jobbágy A., Dunai Z.A., Makra N., Bánvölgyi A., Kiss N., Sárdy M., Sándor S.E., Holló P., Ostorházi E. (2024). Characteristics of the Stool, Blood and Skin Microbiome in Rosacea Patients. Microorganisms.

[47] Jobbágy A., Kiss N., Meznerics F.A., Farkas K., Plázár D., Bozsányi S., Fésűs L., Bartha Á., Szabó E., Lőrincz K. (2022). Emergency Use and Efficacy of an Asynchronous Teledermatology System as a Novel Tool for Early Diagnosis of Skin Cancer during the First Wave of COVID-19 Pandemic. Int. J. Environ. Res. Public Health.

[48] Cantisani C., Ambrosio L., Cucchi C., Meznerics F.A., Kiss N., Bánvölgyi A., Rega F., Grignaffini F., Barbuto F., Frezza F. (2022). Melanoma Detection by Non-Specialists: An Untapped Potential for Triage?. Diagnostics.

[49] Jetter N., Chandan N., Wang S., Tsoukas M. (2018). Field Cancerization Therapies for Management of Actinic Keratosis: A Narrative Review. Am. J. Clin. Dermatol..

[50] Heppt M.V., Trin K., Mille A.-C., Groc M., Delarue A., Bégeault N. (2025). Association Between Local Skin Reactions and Efficacy with 5-Fluorouracil 4% Cream in Actinic Keratosis: A Post-Hoc Analysis of Two Randomised Clinical Trials. Dermatol. Ther..

[51] Heppt M., Steeb T., Leiter U., Berking C. (2019). Efficacy of photodynamic therapy combined with topical interventions for the treatment of actinic keratosis: A meta-analysis. J. Eur. Acad. Dermatol. Venereol..

[52] Pei S., Kaminska E.C.N., Tsoukas M.M. (2017). Treatment of Actinic Keratoses: A Randomized Split-Site Approach Comparison of Sequential 5-Fluorouracil and 5-Aminolevulinic Acid Photodynamic Therapy to 5-Aminolevulinic Acid Photodynamic Monotherapy. Dermatol. Surg..

[53] Boostani M., Pellacani G., Goldust M., Nádudvari N., Rátky D., Cantisani C., Lőrincz K., Bánvölgyi A., Wikonkál N.M., Paragh G. (2025). Diagnosing Actinic Keratosis and Squamous Cell Carcinoma with Large Language Models from Clinical Images. Int. J. Dermatol..

[54] Boostani M., Bánvölgyi A., Zouboulis C.C., Goldfarb N., Suppa M., Goldust M., Lőrincz K., Kiss T., Nádudvari N., Holló P. (2025). Large language models in evaluating hidradenitis suppurativa from clinical images. J. Eur. Acad. Dermatol. Venereol..

[55] Cantisani C., Musolff N., Azzella G., Gargano L., Di Guardo A., Longo C., Guida S., Rossi G., Rovaldi E., Rega F. (2024). Tirbanibulin 1% Ointment Effectiveness for Actinic Keratosis Treatment Evaluated by Dynamic Optical Coherence Tomography. Dermatol. Ther..

[56] Latriglia F., Ogien J., Tavernier C., Fischman S., Suppa M., Perrot J.-L., Dubois A. (2023). Line-Field Confocal Optical Coherence Tomography (LC-OCT) for Skin Imaging in Dermatology. Life.

[57] Stockfleth E., Heppt M.V., Bégeault N., Delarue A. (2023). Evaluating the Efficacy and Safety of 4% 5-Fluorouracil Cream in Patients with Actinic Keratosis: An Expert Opinion. Acta Derm.-Venereol..

[58] Ezzedine K., Painchault C., Brignone M. (2021). Systematic Literature Review and Network Meta-analysis of the Efficacy and Acceptability of Interventions in Actinic Keratoses. Acta Derm. Venereol..

[59] Askew D.A., Mickan S.M., Soyer H.P., Wilkinson D. (2009). Effectiveness of 5-fluorouracil treatment for actinic keratosis—A systematic review of randomized controlled trials. Int. J. Dermatol..

[60] Gupta A., Paquet M. (2013). Network meta-analysis of the outcome ‘participant complete clearance’ in nonimmunosuppressed participants of eight interventions for actinic keratosis: A follow-up on a Cochrane review. Br. J. Dermatol..

[61] Jansen M., Kessels J., Merks I., Nelemans P., Kelleners-Smeets N., Mosterd K., Essers B. (2020). A trial-based cost-effectiveness analysis of topical 5-fluorouracil vs. imiquimod vs. ingenol mebutate vs. methyl aminolaevulinate conventional photodynamic therapy for the treatment of actinic keratosis in the head and neck area performed in the Netherlands. Br. J. Dermatol..

[62] Shoimer I., Rosen N., Muhn C. (2010). Current management of actinic keratoses. Skin Ther. Lett..

[63] Balcere A., Kupfere M.R., Čēma I., Krūmiņa A. (2019). Prevalence, Discontinuation Rate, and Risk Factors for Severe Local Site Reactions with Topical Field Treatment Options for Actinic Keratosis of the Face and Scalp. Medicina.

[64] Singh R., McCain S., Feldman S.R. (2023). Refusal of Retreatment with Topical 5-Fluorouracil Among Patients with Actinic Keratosis: Qualitative Analysis. JMIR Dermatol..

[65] Patel P., Wang J., Bitterman D., Mineroff J., Austin E., Jagdeo J. (2024). Systematic review of randomized controlled trials of topicals for actinic keratosis field therapy. Arch. Dermatol. Res..

[66] Sinclair R., Baker C., Spelman L., Supranowicz M., MacMahon B. (2021). A review of actinic keratosis, skin field cancerisation and the efficacy of topical therapies. Australas. J. Dermatol..

[67] Network NCC (2023). NCCN Clinical Practice Guidelines in Oncology (NCCN Guidelines^®^): Squamous Cell Skin Cancer. Version 2.2023. https://www.nccn.org/guidelines/guidelines-detail?category=1&id=1465.

[68] Holló P., Lengyel Z., Bánvölgyi A., Kiss N. (2024). Diagnosis of Skin Cancer: From the Researcher Bench to the Patient’s Bedside. J. Clin. Med..

[69] Jansen M.H., Kessels J.P., Nelemans P.J., Kouloubis N., Arits A.H., van Pelt H.P., Quaedvlieg P.J., Essers B.A., Steijlen P.M., Kelleners-Smeets N.W. (2019). Randomized Trial of Four Treatment Approaches for Actinic Keratosis. N. Engl. J. Med..

[70] Ahmady S., Jansen M.H.E., Nelemans P.J., Kessels J., Arits A., de Rooij M.J.M., Essers A.B.B., Quaedvlieg P.J.F., Kelleners-Smeets N.W.J., Mosterd K. (2022). Risk of Invasive Cutaneous Squamous Cell Carcinoma After Different Treatments for Actinic Keratosis: A Secondary Analysis of a Randomized Clinical Trial. JAMA Dermatol..

[71] Maarouf M., Kromenacker B.W., Brucks E.S., Hendricks A., Shi V.Y. (2020). Reducing unpleasant side effects of topical 5-Fluorouracil treatment for actinic keratosis: A randomized controlled trial. J. Dermatol. Treat..

[72] EMAE 5-Fluorouracil Topical Application. https://www.ema.europa.eu/en/homepage.

[73] Pierre Fabre Limited Tolerak^®^ 40 mg/g cream. Summary of Product Characteristics. https://www.medicines.org.uk/emc/product/15802/smpc#gref.

[74] Cunningham T.J., Tabacchi M., Eliane J.-P., Tuchayi S.M., Manivasagam S., Mirzaalian H., Turkoz A., Kopan R., Schaffer A., Saavedra A.P. (2017). Randomized trial of calcipotriol combined with 5-fluorouracil for skin cancer precursor immunotherapy. J. Clin. Investig..

[75] Maxwell Regester R., Cannella A.C., Thiele G.M., Griess A.J. (2025). Synergistic Mechanisms of 5-Fluorouracil and Imiquimod in the Treatment of Actinic Keratoses. J. Drugs Dermatol..

[76] Gupta N., Gupta G.D., Singh D. (2022). Localized topical drug delivery systems for skin cancer: Current approaches and future prospects. Front. Nanotechnol..

